# Recommendations to guide sampling effort for polygon-based participatory mapping used to identify perceived ecosystem services hotspots

**DOI:** 10.1016/j.mex.2022.101921

**Published:** 2022-11-11

**Authors:** Rachael H. Carrie, Lindsay C. Stringer, Thi Van Hue Le, Nguyen Hong Quang, Christopher R. Hackney, Van Tan Dao, Thi Thanh Nga Pham, Claire H. Quinn

**Affiliations:** a Sustainability Research Institute, School of Earth and Environment, University of Leeds, Leeds LS2 9JT, United Kingdom; b Central Institute for Natural Resources and Environmental Studies, Vietnam National University, 19 Le Thanh Tong Street, Hoan Kiem, Ha Noi, Viet Nam; c Vietnam National Space Center (VNSC), Vietnam Academy of Science and Technology (VAST), 18 Hoang Quoc Viet, Hanoi 100000, Viet Nam; d Energy and Environment Institute, University of Hull, Hull HU6 7RX, United Kingdom; e Mangrove Ecosystem Research Center, Hanoi National University of Education, 136 Xuan Thuy, Cau Giay, Ha Noi, Viet Nam

**Keywords:** Participatory GIS (PGIS), Public participation GIS (PPGIS), Polygon overlap, Spatial agreement, Triangulation

## Abstract

Participatory mapping is increasingly used to map spatial variation in people's perceptions about ecosystem services. It has growing use in the identification of locations where places perceived to be important converge. Few recommendations have been published to navigate decisions about sampling effort in participatory mapping research when polygon data is collected, although one recommendation is for ≥ 25 participants assuming each participant maps c. 4–5 polygons per ecosystem service. Underlying data informing this recommendation reflects a particular context: collected using postal questionnaires to map a vast spatial area in southern Australia. Although not intended as definitive or suited to all contexts, the 25 participant (or 100-125 polygon) minimum sometimes informs participatory mapping research. Our empirical work, undertaken using face-to-face questionnaires in a small Vietnamese coastal study area, suggests the recommendation may not be appropriate in all contexts. We propose a modified stepwise approach which:

• Prioritises spatial agreement (polygon overlap) rather than polygon count and participant numbers to assess data sufficiency
• Uses narratives to triangulate outputs generated from participatory mapping data to reduce uncertainty related to low polygon counts

## Graphical abstract

A graphical abstract is mandatory. The graphical abstract should summarize the contents of your article in a concise, pictorial form. Authors must provide images that clearly represent the work described in the article. Graphical abstracts should be submitted as a separate file. Image size: *please provide an image with a minimum of 531 × 1328 pixels (h × w) or proportionally more***.** The image should be readable at a size of 5 × 13 cm using a regular screen resolution of 96 dpi. Preferred file types: TIFF, EPS, PDF or MS Office files.

**Figure:**
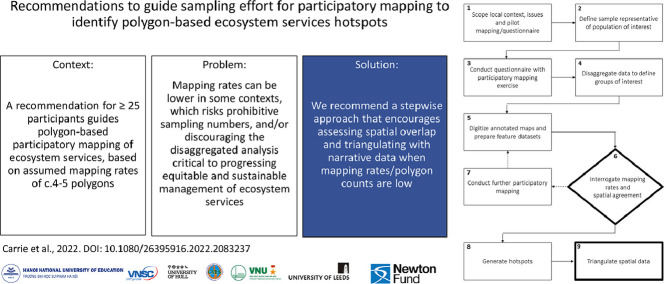

Specifications table

**Table:** 

Subject area:	Environmental Science
More specific subject area:	Participatory/Public Participation Geographic Information Systems (PGIS/PPGIS)
Name of your method:	Recommendations to guide sampling effort for polygon-based participatory mapping to identify perceived ecosystem services hotspots
Name and reference of original method:	Brown, G. G. & Pullar, D. V. (2012). An evaluation of the use of points versus polygons in public participation geographic information systems using quasi-experimental design and Monte Carlo simulation. International Journal of Geographical Information Science, 26(2), 231–246. https://doi.org/10.1080/13658816.2011.585139
Resource availability:	N/A

## Method details

### Rationale

Participatory mapping is commonly used to map spatial variation in social perceptions about ecosystem services [1]. This method is seeing growing utility in identifying areas perceived to provide ecosystem services, including those areas perceived to be of greater importance for ecosystem services than others [2], [3], [4] (hereafter referred to as ‘hotspots’). There are numerous methodological choices to be made when using participatory mapping, such as an individual or group unit of analysis, the type of feature used to locate the attribute of interest: point, polygon and/or line, the mode of delivery: e.g. face-to-face vs postal or online questionnaire, and the amount of data to collect [1,5]. Sampling effort is important in participatory mapping because of spatial data sufficiency concerns [1,5] which may be distinct from concerns related to population inference. To navigate this decision in participatory mapping research when polygon data is collected from individuals, Brown & Pullar [6] identified as a heuristic guiding principle “*a minimum of 25 respondents for polygon-based PPGIS systems assuming 4–5 polygons identified per attribute per respondent on average*” (p244). Given this recommendation is based on mapping rates of 4-5 polygons per participant, the actual methodological requirement is for an average of 100-125 polygons per mapped attribute. This range was determined by evaluating polygon geometry in comparison to points to understand the amount of data needed for attributes identified using the two methods to converge on a collective spatial ‘truth’. Although the authors cautioned that the recommended range was not definitive, nor intended as universally prescriptive, a minimum sample size of 25 participants often informs participatory mapping study design when polygons are used [e.g. [7], [8], [9], [10], [11], [12]]. Yet, context can influence the number of polygons drawn by those mapping, e.g. due to the amount and relative spatial arrangement of ecosystems in the study area, and spatial scale [13,14].

Brown & Pullar's [6] recommendation was based on data reflecting a particular context: collected using postal questionnaires to locate values associated with a vast spatial area, the Otways coastal region of Victoria, Australia comprising coast, hinterlands and plains [15]. Research we conducted using face-to-face questionnaires over a smaller spatial area (c.20km^2^) focused on one ecosystem type (mangroves) in northern Vietnam, and achieved lower mapping rates. Some other polygon-based studies conducted over smaller spatial areas (e.g. <100km^2^) in different contexts also report mapping rates lower than the recommended 4-5 markers per attribute per respondent [e.g. 16,17]. This reiterates the caution expressed that the 25 participant recommendation may not be relevant to participatory mapping undertaken in all contexts [6], and raises the possibility that lower mapping rates may sometimes be a feature when mapping is conducted in small study areas.

Our mapping rates suggested 40-80% (300-615) of households in our study villages would need to be sampled to achieve at least 100-125 polygons per attribute for the ecosystem services considered most important by study participants. Such sample numbers place a prohibitive burden on participating villages and research budgets in locations where face-to-face mapping offers the most effective data collection tool. Resource needs and costs may also discourage wider use and adoption of participatory mapping by practitioners to inform policy and management decisions: a critical issue participatory mapping research hopes to overcome [18]. They also risk discouraging disaggregated ecosystem services research because 100-125 polygons would be required per attribute, per disaggregated group, and may inadvertently encourage data aggregation to achieve polygon counts, for example by collapsing individual ecosystem services into broader categories. These issues have far reaching implications, particularly where smaller-scale studies pay attention to, and their findings are of great relevance for, local stakeholders that rely directly on ecosystem services [19,20]. It is also pertinent where approaches that can more easily achieve large sample numbers, such as postal questionnaires and web-based surveys, may be less effective. It is locations with these characteristics where least ecosystem services PGIS research has been published [1], and where low polygon counts may also provide important insights.

In their guidance paper, Brown & Pullar [6] emphasize that “*more spatial agreement among respondents equates to higher confidence in place attributes*” (p244). Spatial agreement in polygon-based participatory mapping is often measured by counting the number of times polygons overlap (as done by Brown & Pullar [6] and for example [[2], [3], [4],^7^]). Our experience indicates spatial agreement measured by polygon overlap may provide a more useful guide than participant or polygon numbers. It also underlines the utility of narrative data, both to triangulate spatial data and to enable perceptions about specific places to be better understood. We outline a stepwise approach that enables consideration of spatial agreement and data triangulation to overcome potential issues associated with data uncertainty when mapping rates are low.

## Approach

**Fig. 1:**
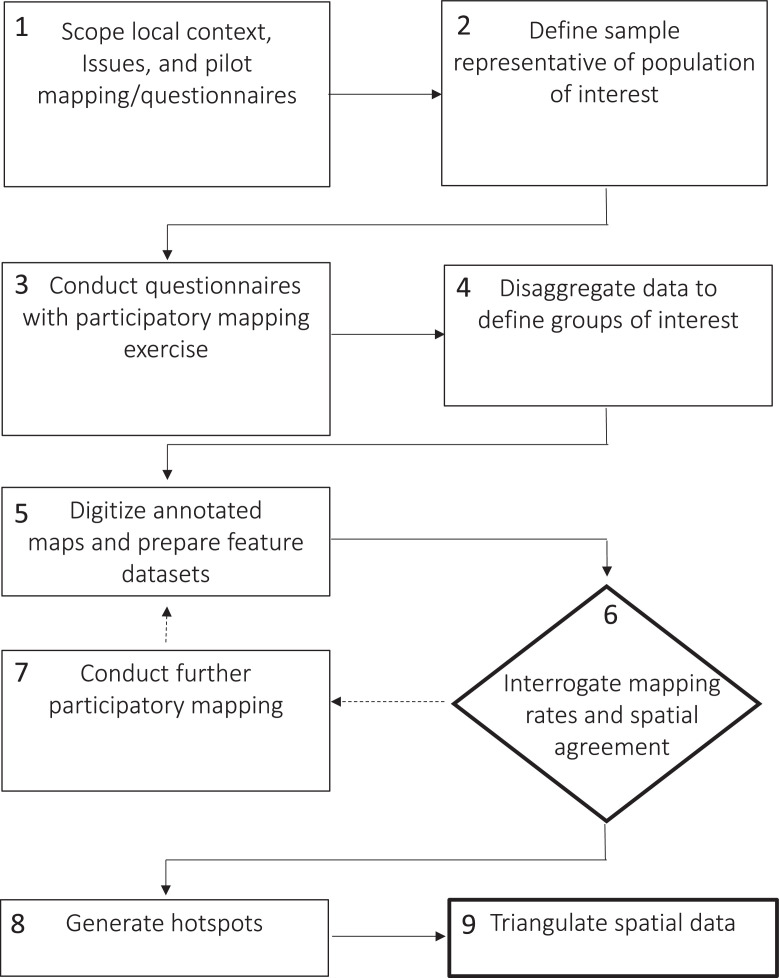
A stepwise approach to polygon-based participatory mapping to generate ecosystem services hotspots based on overlap of polygons reflecting areas of perceived importance. Steps 6 and 9 (bold) are additions to what we perceive to be a typical approach. Step 6 is contained with a diamond to reflect the need for a decision.

Fig. 1 presents a stepwise approach for the purposes of disaggregated analysis. It reflects our interpretation of a generalized approach to the simple assessment of spatial association using polygon overlap counts based on participatory mapping conducted using questionnaires, with the addition of two steps to overcome the issues identified above. It reflects our experiences of administering questionnaires face-to-face. We outline the approach below before illustrating it in detail drawing on our research.

Step 1 involves scoping the study area to understand context and identify study boundaries, attributes being mapped, local landmarks for quality control, and to pilot appropriate language and mapping approaches. Step 2 uses outputs from the scoping work to enable a sample representative of the population of research interest to be defined. Step 2 is not specific to participatory mapping research, but typical of procedures followed in social science research generally.

In step 3, a household questionnaire that includes a participatory mapping exercise is administered. The design of the questionnaire and participatory mapping is informed by the outputs of the scoping exercise conducted in step 1.

Step 4 involves disaggregating the sample into interest groups identified *a priori*, or by analysing non-spatial data collected during household surveys (step 3). The approach taken, data and analysis used, and groups identified will be determined by the research question. We illustrate this in the section below in relation to a research question linked to capacity to adapt to change in mangrove ecosystem services perceived to be important by coastal households.

In step 5, annotated maps drawn by respondents are digitised and the spatial dataset separated into feature classes for each attribute being mapped for each disaggregated group.

Step 6 is a modification to what we perceive to be a typical approach to polygon-based participatory mapping when ecosystem services hotspots are generated by counting overlap in polygons reflecting areas of perceived importance. This step involves calculating mapping rates and polygon overlap. If mapping rates are low, polygon overlap can be explored and a decision made to undertake further mapping work (see step 7), or to proceed with hotspot generation (see step 8) with the knowledge that triangulation of spatial data with narrative information may be required. Importantly, step 6 can and should be done before hotspots are generated to avoid biasing the decision based on results.

In Step 8, hotspots are generated to identify the locations with greatest polygon overlap following methods such as that outlined by Brown and Pullar [6] and others [e.g. 8,23].

In step 9 narrative data can be analysed to provide a means of triangulation. Such data might be collected using open-ended survey questions at the time of participatory mapping, or subsequently using interviews.

## Approach validation

To illustrate our approach, we present our research conducted during July-December 2018 and July 2019 that aimed to assess if and why perceptions about ecosystem services and service providing places vary among households with different capacities to adapt to change in ecosystem service supply. Our study area was a mangrove system in the Red River Delta, Northern Vietnam, covering approximately 20 km^2^. Full details of the study location, the three adaptive capacity household groups, the ecosystem services mapped, and methods are provided by Carrie et al., [21].

Step 1: Focus groups and transect walks were used to identify the boundary of our study mangrove system, develop a standardized list of ecosystem services to be mapped, and identify indicators to use to disaggregate our sample into different adaptive capacity groups. Outputs from these activities informed the development of a questionnaire and associated paper-based participatory mapping exercise. These were piloted with 30 households to ensure phrasing and content were contextually relevant, and to explore the use of point and polygon mapping approaches. Participants expressed concern about using points to depict areas important to them. A particular issue centred on determining how far apart points should be drawn when discrete locations were not used. For example, the ecosystem services ‘opportunities for physical experiences’ and ‘food’ collection often represented continuous movement. Thus, respondents felt it more accurate to draw shapes (polygons) around the areas perceived to be important.

Step 2: Scoping revealed that three (of nine) villages in the study area were currently or historically located adjacent to the coastline. We purposively selected these villages to sample participants from as households there were most likely to have developed links with the mangrove system over time. We surveyed 150 households in total, to representing approximately 16% of households in each study village. Households were selected at random by choosing every *n*th house from village household lists. The sample size represented a balance between obtaining a representative sample of the study villages [22], availability of research resources, and minimizing the burden to participants. We also assumed this sample size would be sufficiently large to enable disaggregated spatial analysis given the ≥25 participant recommendation [6].

Step 3: A finalized household questionnaire with a laminated colour map at scale 1:15,000 (downloaded from Google Earth) was administered face-to-face with each sampled household. To increase confidence in the accuracy of the locations identified, map literacy was checked first. Respondents were orientated by locating their homes on the map and requested to identify local landmarks. Three households were unable to locate landmarks, so did not complete the mapping exercise. Those able to locate landmarks were requested to draw polygons in locations perceived to be important for ecosystem services to their household. There were no restrictions on the number of locations participants could identify, nor the size or shape of polygon.

Step 4: We disaggregated our sample using non-linear principal component analysis to identify key axes of variation in the adaptive capacity data, and cluster analysis on principal component object scores for each household. This identified three distinct groups of households with different types and levels of adaptive capacity: 1 – *accumulating* households; 2 – *coping* households; and 3 – *flexible* households.

Step 5. Twelve ecosystem services were identified as providing benefits to households, and similar ecosystem services were identified most frequently by all three groups (Table 1).

**Table 1:** Ecosystem services identified as important for providing benefits to households with different adaptive capacities in a mangrove system in the Red River Delta, Vietnam. Shaded cells indicate ecosystem services identified by at least two thirds of all groups, and for which we conducted hotspot analysis.

Ecosystem Service	IPBES Reporting Category [29]	No. of participants identifying ecosystem services as important to their household. Percentage of sample is provided in parentheses		
		Accumulating households	Coping households	Flexible households
Protection from storms and erosion	Regulation of hazards and extreme events	44 (100)	37 (100)	69 (100)
Food	Food and feed	31 (71)	35 (95)	65 (94)
Sediment accumulation	Formation, protection and decontamination of soils and sediments	34 (77)	29 (78)	57 (83)
Habitat provisioning	Habitat creation and maintenance	37 (84)	26 (70)	56 (81)
Recreation	Physical and psychological experiences	34 (77)	22 (60)	51 (74)
Climate regulation	Regulation of climate	24 (55)	19 (51)	35 (51)
Air quality	Regulation of air quality	21 (48)	13 (35)	33 (48)
Learning	Learning and inspiration	12 (27)	3 (8)	23 (33)
Medicine	Medicinal, biochemical and genetic resources	31 (71)	16 (43)	43 (62)
Identity	Supporting identities	4 (9)	3 (8)	5 (7)
Water quality	Regulation of freshwater and coastal quality	8 (18)	2 (5)	12 (17)
Energy	Energy	2 (5)	1 (3)	3 (4)

In total, 628 polygons showing the areas perceived to be important for ecosystem services were digitized from 147 annotated maps using ArcMap, version 10.4 (Environmental Systems Research Institute, Redlands, California). Digitized polygons were organized by the perceived ecosystem services and adaptive capacity group membership in a file geodatabase. Mapping frequencies, mapping rates and polygon overlap data are presented in Table 2.

**Table 2:** Ecosystem services mapping data for groups of households with different adaptive capacities in a mangrove system in the Red River Delta, Vietnam. Shaded cells indicate occasions where <25 participants drew polygons to identify ecosystem service places perceived important to their households. Only ‘protection from storms and erosion’ was identified by > 25 participants in all groups. Overlap ranges highlighted in bold are within those published by others when >100 polygons were drawn [4,8,24].

Ecosystem Service	No. of participants mapping specific ecosystem services places^a^. Mapping rates are provided in parentheses			Maximum number of overlapping polygons. Number of polygons drawn is provided in parentheses. Note that <100 polygons were drawn on all occasions		
	Accumulating households	Coping households	Flexible households	Accumulating households	Coping households	Flexible households
Protection from storms and erosion	33 (1.0)	29 (1.38)	51(1.02)	**29 (33)**	**25 (40)**	**41 (52)**
Food	8 (1.0)	5 (1.0)	33 (1.09)	6 (8)	4 (5)	**29 (36)**
Sediment accumulation	32 (1.0)	24 (1.04)	47 (1.0)	**18 (32)**	**13 (25)**	**27 (47)**
Habitat provisioning	26 (1.04)	18 (1.11)	43 (1.05)	**22 (27)**	**14 (20)**	**30 (45)**
Recreation	33 (1.27)	22 (1.32)	51 (1.20)	**21 (42)**	**16 (29)**	**41 (61)**
Climate regulation	10 (1.0)	7 (1.0)	16 (1.0)	**10 (10)**	7 (7)	**15 (16)**
Air quality	6 (1.0)	5 (1.20)	13(1.0)	5 (6)	4 (6)	**13 (13)**
Learning	10 (1.20)	2 (1.0)	18 (1.11)	6 (12)	0 (2)	**14 (20)**
Medicine	0 (-)	2 (1.0)	5 (1.20)	0 (0)	2 (2)	3 (6)
Identity	4 (1.0)	1 (1.0)	4 (1.0)	1 (4)	0 (1)	2 (4)
Water quality	4 (1.0)	1 (1.0)	7 (1.0)	4 (4)	0 (1)	5 (7)
Energy	2 (1.0)	1 (1.0)	2 (1.0)	2 (2)	0 (1)	2 (2)

a Excluding polygons that covered >50% of the total area.

Step 6: Interrogation of the spatial data revealed <25 participants drew polygons on 26 of 36 occasions (where an occasion represents an ecosystem service in an adaptive capacity group), and that ≥25 participants in all three adaptive capacity groups drew polygons for only one ecosystem service (protection from storms and erosion) (Table 2). Mapping rates were calculated for each ecosystem service within each adaptive capacity group by dividing the number of polygons by the number of participants drawing them. Rates averaging between 1 and 1.38 polygons depending on the ecosystem service mapped (Table 2) were considerably lower than the average 4-5 polygon mapping rate used by Brown & Pullar [6] when developing their recommendation. Consequently, our polygon counts were below the recommended minimum of 100 polygons on every single occasion (Table 2).

Aggregating our adaptive capacity groups would have achieved ≥25 participants for seven of the 12 ecosystem services, or at least 100 polygons for three ecosystem services. However, it would also have resulted in the loss of considerable detail and nuance, and would ultimately have prevented us from achieving our research aim.

We counted polygon overlaps using the approach developed by Martinez in [23], which was similar to the approach used by Brown and Pullar [6]. During the process of trying to understand why our mapping rates were comparatively low, we noted that the maximum number of overlapping polygons in our data revealed spatial agreement similar to that identified when at least 100 polygons were drawn in other published data on 17 of the 36 occasions (Table 2). For example, the maximum number of overlapping polygons ranged between 10 and 38 for attributes for which at least 100 polygons were mapped in a coastal study area of approximately 60,000 km^2^ in Kimberley, Australia [8,24], and between 29 and 39 in a multiple use landscape in Iran covering 5,272km^2^ [4].

Step 7: Given levels of spatial agreement, we proceeded with the original disaggregated analysis focusing on the four ecosystem services identified as those most important by all groups: protection from storms and erosion, food, sediment accumulation and habitat provisioning (Table 1). Three of these achieved polygon overlap in the range observed in the Kimberley study [8,24]. However, one ecosystem service (food) achieved lower polygon overlap for two of the adaptive capacity groups (Table 2). We incorporated questions within already planned interviews to better understand and triangulate participatory mapping data for food and other ecosystem services.

Step 8: We identified hotspots using cut-off values at two commonly used levels of conservatism: the top 67% and 90% of overlap values.

Step 9: Interviews were already part of our research methodology because we wanted to analyse narratives to explore strategies used by different households to adapt to change in mangrove systems and ecosystem service places over time. Narrative data, produced for example from focus group discussions, interviews or open-ended survey questions, is often used to explore people's relationships with ecosystem services [25], [26], [27], including in combination with participatory mapping. For instance, used with mapping data, narratives have identified drivers of change to ecosystem service supply [3], provided supplementary data of a sensitive nature [28], insights about why places were not mapped, and identified interlinkages between attributes mapped and development preferences [10]. To prompt discussion about places mapped/not mapped and why that was so, our interviews were structured around the maps annotated by the household in question. One intention was to supplement qualitative data collected during the mapping exercise using open-ended questions with longer narratives to triangulate spatial data and consider uncertainty produced by low mapping rates and polygon counts.

### Mapping rates

Following the logic underpinning difficulties identifying discrete locations while piloting point and polygon mapping approaches (see step 1), many respondents found it problematic to determine where one polygon ended and another began in areas of continuous land cover. Most preferred to draw one polygon to cover the entire area important to them, rather than more numerous, connected polygons. It seems logical to assume that a larger study area might include more diverse land cover or uses or spatially dispersed patches of the ecosystem of interest, and that respondents may map more places.

### Polygon counts

The lower number of polygons drawn in our study arose for reasons other than low mapping rates. Not all respondents identifying ecosystem services as important to their household mapped ecosystem service places. Some respondents were unable to identify specific places. Of those, many instead indicated that large areas of the system were important. We excluded from the analysis polygons covering >50% of the total area, since research suggests they contribute little to defining hotspots [6].

Discrepancies in the numbers of those identifying an ecosystem service as important and identifying a linked ecosystem service place, were not entirely due to exclusion of polygons >50% of the total area. Not all respondents were able to indicate a location for all of the ecosystem services they considered important. Of the four ecosystem services which formed the focus of our analysis, this was particularly the case for food. Comparatively few *coping* and *accumulating* households that considered this ecosystem service important, drew polygons for it (Table 2). Including time for discussion in interviews to determine why the numbers of polygons drawn was not higher helped us to understand why food providing places were mapped at lower frequency. *Coping* households described that although the ecosystem service was still important to their households, their ability to access perceived food providing places had become reduced over time. *Accumulating* households explained how the acquisition of aquaculture ponds provided them with alternative, more convenient sources of similar food items. These insights about low polygon counts were as important to our study as those about ecosystem service places with ‘sufficient’ polygon counts and overlap, and emphasise why it can be particularly valuable to incorporate narrative data within participatory mapping research.

### Hotspot triangulation

Interview responses and answers to open-ended questions asked during the mapping exercise were analysed inductively to identify specific features located at hotspots and if, how and why they differed between groups. To increase our confidence in the differences identified by the spatial data, we wanted to see clear differences between hotspot locations and features present there. Taking habitat provisioning as an example, the key difference in hotspots identified at the 67% cut-off was linked to the presence of aquaculture ponds. Hotspots for *flexible* and *coping* households included areas within ponds; hotspots for *accumulating* households did not. *Flexible* and *coping* households mapping these places explained that remnant old growth trees were still present in some ponds and that these older trees provided habitat for the birds they liked to observe or listen to. A*ccumulating* households spoke about trees outside of ponds as more important for birds. *Accumulating* households owned or leased these ponds, and for habitat provisioning they also provided descriptions of places where plankton became trapped in mangrove trees after flowing in with the tide, and how sea animals used these places to feed before travelling to naturally stock ponds. The areas identified coincided with the habitat provisioning hotspots generated for this group, located adjacent to but outside of ponds.

Narrative descriptions such as these increased our confidence in the different places identified as hotspots. Even for food, for which spatial agreement was low for *coping* and *accumulating* households groups, triangulation increased confidence and helped us to understand the reasons ecosystem service places were not mapped. *Coping* households were characterized by an older demographic and included people less physically able and with fewer assets. The hotspots generated for this group coincided with areas identified as easiest to access because they had smaller channels, less dense mangrove growth, and were in close proximity to entry points. In contrast, hotspots produced from polygons drawn by *accumulating* households covered the larger channels they described accessing using boats, and the mangrove forest located on either side.

Our study highlights that mapping rates can vary and suggests there is unlikely to be a ‘one-size fits all’ recommendation for minimum participant numbers based on them. Incorporating steps in the research process to quantify and interrogate mapping rates prior to the generation of hotspots enables action to be taken to increase data confidence, either by undertaking additional mapping with the aim of increasing spatial agreement, or by conducting explanatory qualitative research. Incorporation of interviews at the end of the research process, although probably less burdensome than increasing the number of participatory mapping participants, may still be problematic from budgetary or time perspectives and/or because of participant fatigue. At the very least, open-ended questions asking why particular locations were mapped and/or not mapped could be included in the participatory mapping exercise to facilitate triangulation of hotspots.

We have not outlined concrete numerical recommendations to guide decisions in our approach. If such recommendations are made without understanding how they may vary in different contexts, we risk perpetuating the issue we tackle here. Our recommendations include being mindful of participant numbers, but to also consider mapping rates and spatial overlap. Table 2 provides an indication of the range in numbers of overlapping polygons that might inform this consideration based on the few studies found that published this data type. While such flexibility may be considered a potential limitation of our method, we also contend that prescriptive numbers may not be appropriate to all circumstances. Any limitation this creates could be mitigated to some extent through the analysis of narrative data to triangulate spatial information about places perceived important for ecosystem services.

## Ethics statements

This research was approved by the ESSL, Environment and LUBS (AREA) Faculty Research Ethics Committee at the University of Leeds (AREA 17-112). Informed consent was obtained from all participants prior to data collection.

## CRediT authorship contribution statement

**Rachael H. Carrie:** Conceptualization, Data curation, Methodology, Investigation, Formal analysis, Visualization, Writing – original draft, Writing – review & editing. **Lindsay C. Stringer:** Methodology, Writing – review & editing, Funding acquisition. **Thi Van Hue Le:** Methodology, Investigation, Writing – review & editing, Funding acquisition. **Nguyen Hong Quang:** Formal analysis, Writing – review & editing. **Christopher R. Hackney:** Writing – review & editing, Funding acquisition. **Van Tan Dao:** Writing – review & editing, Funding acquisition. **Thi Thanh Nga Pham:** Writing – review & editing, Funding acquisition. **Claire H. Quinn:** Methodology, Writing – review & editing, Funding acquisition.

## Declaration of Competing Interests

The authors declare that they have no known competing financial interests or personal relationships that could have appeared to influence the work reported in this paper.

## Data Availability

The data that support the findings of this study are available in Reshare at https://reshare.ukdataservice.ac.uk/854718/.
